# Tyrosylprotein sulfotransferase-dependent and -independent regulation of root development and signaling by PSK LRR receptor kinases in Arabidopsis

**DOI:** 10.1093/jxb/erab233

**Published:** 2021-05-24

**Authors:** Christine Kaufmann, Nils Stührwohldt, Margret Sauter

**Affiliations:** 1 Plant Developmental Biology and Physiology, University of Kiel, Kiel, Germany; 2 University of Antwerp, Belgium

**Keywords:** Arabidopsis, atrichoblast, phytosulfokine, root development, root hair, sulfated peptide signaling, transcriptome, tyrosylprotein sulfotransferase

## Abstract

Tyrosine-sulfated peptides are key regulators of plant growth and development. The disulfated pentapeptide phytosulfokine (PSK) mediates growth via leucine-rich repeat receptor-like kinases, PSKR1 and PSKR2. PSK receptors (PSKRs) are part of a response module at the plasma membrane that mediates short-term growth responses, but downstream signaling of transcriptional regulation remains unexplored. In Arabidopsis, tyrosine sulfation is catalyzed by a single-copy gene (*TPST*; encoding tyrosylprotein sulfotransferase). We performed a microarray-based transcriptome analysis in the *tpst-1* mutant background that lacks sulfated peptides to identify PSK-regulated genes and genes that are regulated by other sulfated peptides. Of the 169 PSK-regulated genes, several had functions in root growth and development, in agreement with shorter roots and a higher lateral root density in *tpst-1*. Further, *tpst-1* roots developed higher numbers of root hairs, and PSK induced expression of *WEREWOLF* (*WER*), its paralog *MYB DOMAIN PROTEIN 23* (*MYB23*), and *At1g66800* that maintain non-hair cell fate. The *tpst-1 pskr1-3 pskr2-1* mutant showed even shorter roots, and higher lateral root and root hair density than *tpst-1*, revealing unexpected synergistic effects of ligand and PSKR deficiencies. While residual activities may exist, overexpression of *PSKR1* in the *tpst-1* background induced root growth, suggesting that PSKR1 may be active in the absence of sulfated ligands.

## Teaser

Phytosulfokine (PSK) receptor signaling promotes root elongation, determines lateral root density, and maintains non-hair cell fate partially independent of TPST responsible for the activating sulfation of PSK.

## Introduction

Post-translationally modified peptides are an emerging class of signaling molecules that are involved in regulation of plant growth and development, and in responses to abiotic and biotic stresses including pathogen attacks. Post-translationally modified peptides are derived from larger pre-proproteins that require several steps of proteolytic cleavages. Besides proteolytic processing to release the peptide moiety from the inactive precursor, they may rely on tyrosine sulfation, proline hydroxylation, and arabinosylation of hydroxyprolines to receive full peptide activities (Matsubayashi, 2014; Kaufmann and Sauter, 2019; Stührwohldt and Schaller, 2019).

Tyrosine sulfation of signaling peptides is catalyzed by the Golgi-localized tyrosylprotein sulfotransferase (TPST) that is encoded by a single-copy gene in Arabidopsis (Komori *et al.*, 2009). Several classes of sulfated peptides have so far been identified, namely phytosulfokine (PSK), plant peptides containing sulfated tyrosine (PSYs), root meristem growth factors [RGFs/Golven (GLV)/CLE-like (CLEL)], and Casparian strip integrity factors (CIFs) that include Twisted Seed 1 (TWS1) (Doll *et al.*, 2020; Okuda *et al.*, 2020). For most of them, including PSK, experimental data indicated that tyrosine sulfation is required for full peptide activity (Matsubayashi and Sakagami, 1996; Matsubayashi *et al.*, 2006; Amano *et al.*, 2007; Kutschmar *et al.*, 2009; Matsuzaki *et al.*, 2010; Meng *et al.*, 2012; Whitford *et al.*, 2012; Fernandez *et al.*, 2013; Doblas *et al.*, 2017; Nakayama *et al.*, 2017; Okuda *et al.*, 2020).

TPST loss-of-function mutants are an important tool to analyze functions of and processes triggered by tyrosine-sulfated peptides. Several mutants have been identified, all lacking activity of TPST and consequently activities of tyrosine-sulfated peptides (Komori *et al.*, 2009; Zhou *et al.*, 2010; Kang *et al.*, 2014). *tpst* mutants, known as *tpst-1*, *active quiescent center* (*aqc1-1* to *aqc1-3*), and *hypersensitive to Pi starvation7* (*hps7*), are characterized by an overall dwarfed phenotype, pale-green leaves, and stunted roots which are brought about by defective maintenance of the root stem cell niche, decreased meristematic activity, decreased cell expansion (Komori *et al.*, 2009; Zhou *et al.*, 2010; Kang *et al.*, 2014), and hypersensitivity to fructose (Zhong *et al.*, 2020). TPST acts to maintain the root stem cell niche by regulating basal and auxin-induced expression of the transcription factors Plethora 1 and 2 (PLT1 and PLT2) (Zhou *et al.*, 2010).

PSK is one of the most extensively studied sulfated peptides with regards to physiological functions. Mature PSK is a disulfated pentapeptide of the sequence Y(SO_3_H)-I-Y(SO_3_H)-T-Q that is derived from precursor proteins that vary in length from 77 to 109 amino acids in Arabidopsis (Kaufmann and Sauter, 2019). Unlike all other sulfated peptides, PSK precursor proteins share a fully conserved sequence of the mature pentapeptide, with PSK6, that differs in the last amino acid of the PSK pentapeptide, as an exception. However, since no ESTs have been reported and expression levels according to RNA-seq data are extremely low, *PSK6* is considered to be a pseudogene (Kaufmann and Sauter, 2017).

The enzymes that are responsible for precursor processing to release the PSK pentapeptide are largely unknown. The subtilisin-like serine protease 1.1 (SBT1.1) was shown to cleave the Arabidopsis PSK4 precursor peptide *in vitro* (Srivastava *et al.*, 2008). However, the cleavage site is N-terminal of the mature peptide within the variable part of the precursor, and the physiological relevance of this cleavage and of SBT1.1 for PSK biogenesis remains unclear. In tomato, an aspartate-specific SBT (tomato phytaspase 2, *Sl*Phyt2) was shown to be involved in PSK maturation (Reichardt *et al.*, 2020). The eight PSK precursors in tomato (Reichardt *et al.*, 2020) and the seven PSK precursors in Arabidopsis (Kaufmann and Sauter, 2019) share an aspartate residue on the amino side of the PSK pentapeptide. However, the Arabidopsis protease cleaving the N-terminal Asp has not been identified to date. An unusual precursor protein is PSK1 that is flanked by Asp on both sides of the mature peptide. We could recently show that the C-terminal Asp is cleaved by Arabidopsis SBT3.8 to release the active C-terminus (Stührwohldt *et al.*, 2021).

PSK was originally identified as a growth factor that promotes cell division of asparagus cells grown in culture at low density (Matsubayashi and Sakagami, 1996), and has since been linked to multiple physiological functions. In plant reproductive processes, PSK promotes pollen germination (Chen *et al.*, 2000) and pollen tube elongation (Stührwohldt *et al.*, 2015), and it guides the pollen tube from the transmitting tract along the funiculus to the embryo sac to support seed production (Stührwohldt *et al.*, 2015). Further, PSK induces root, hypocotyl, and leaf growth mainly by promoting cell expansion (Kutschmar *et al.*, 2009; Stührwohldt *et al.*, 2011; Hartmann *et al.*, 2014). Cotton fiber elongation is also driven by enhanced cell elongation and promoted by overexpression of a putative *GhPSK* gene (Han *et al.*, 2014). In addition, PSK differentially affects plant immunity and stress responses. It supports the response to the necrotrophic fungi *Alternaria brassicicola* and *Sclerotinia sclerotiorum* and the bacterium *Ralstonia solanacearum*, and it represses the response to hemi-/biotrophs such as *Pseudomonas syringae* and the oomycete *Hyaloperonospora arabidopsidis* (Loivamäki *et al.*, 2010; Igarashi *et al.*, 2012; Mosher *et al.*, 2013; Rodiuc *et al.*, 2016). Zhang *et al.* (2018) showed that PSK signals the auxin-dependent immune responses in tomato after infection with the necrotrophic fungus *Botrytis cinerea*. Recent studies also revealed a role for PSK signaling in osmotic and drought stress adaptation (Rajamanickam *et al.*, 2021, Preprint; Stührwohldt *et al.*, 2021).

The major understanding of PSK signaling comes from the identification of the plasma membrane-localized PSK receptors PSKR1 and PSKR2, that belong to the large family of leucine-rich repeat receptor-like kinases (LRR-RLKs). PSK binds extracellularly to the island domain of PSKR1 and PSKR2 located between the LRRs (Matsubayashi *et al.*, 2006; Wang *et al.*, 2015). The intracellular PSKR1 domain functions as an essential kinase (Irving *et al.*, 2012; Hartmann *et al.*, 2014, 2015; Kaufmann and Sauter, 2017). At defined Ser, Thr, and Tyr residues, the PSKR1 kinase autophosphorylates (Hartmann *et al.*, 2015; Mitra *et al.*, 2015; Muleya *et al.*, 2016; Kaufmann and Sauter, 2017). PSKR1 forms a heterodimer together with the promiscuous co-receptors SERK1, SERK2, and BAK1/SERK3 (Ladwig *et al.*, 2015; Wang *et al.*, 2015), directly interacts with the H^+^-ATPases AHA1 and AHA2, and forms a functional complex with cyclic nucleotide‐gated channel 17 (CNGC17) (Ladwig *et al.*, 2015).

While the response module consisting of PSKRs, BAK1, AHA1, AHA2, and CNGC17 induces PSK-mediated growth, only a few downstream components of PSK signaling are known (Zhang *et al.*, 2018). To identify new players in PSK signaling, we set up a microarray approach to identify PSK-regulated genes and genes that are regulated by other sulfated peptides by using the *tpst-1* mutant as a tool. The transcriptome data prompted a more detailed analysis of root development. The results revealed a role for PSKR signaling in determining lateral root density, and in maintaining non-hair cell fate by regulating the transcription factor genes *WEREWOLF* (*WER*), its paralog *MYB DOMAIN PROTEIN 23* (*MYB23*), and *At1g66800* (Lee and Schiefelbein, 1999; Matsui *et al.*, 2005; Deal and Henikoff, 2010). The characterization of a *tpst-1 pskr1-3 pskr2-1* triple mutant revealed unexpected synergistic effects of TPST deficiency and PSKR deficiency that may suggest activity of PSKRs dependent on and independent of their ligand PSK.

## Materials and methods

### Growth conditions and plant material

All experiments were carried out with *Arabidopsis thaliana* ecotype Col-0. The T-DNA insertion line *tpst-1* (SALK_009847) and the double knockout line *pskr1-3 pskr2-1* were described previously (Komori *et al.*, 2009; Kutschmar *et al.*, 2009; Stührwohldt *et al.*, 2011). The triple knockout line *tpst-1 pskr1-3 pskr2-1* was generated by crossing *tpst-1* with *pskr1-3 pskr2-1*. Loss of all three transcripts was verified by semi-quantitative RT-PCR. Seeds were surface-sterilized in 2% (v/v) sodium hypochlorite for 15 min, washed five times with autoclaved water, and subsequently laid out on 0.5× Murashige and Skoog medium (Duchefa, Harlem, The Netherlands) with 1.5% (w/v) sucrose, solidified with 0.4% (w/v) Gelrite (Duchefa). If indicated, media were supplemented with 1 µM PSK (Pepscan, Lelystad, The Netherlands). For growth on soil, plants were grown in a 2:3 sand:humus mixture that was frozen at –80 °C for 2 d to avoid insect contamination, and watered regularly with tap water. After 2 d of stratification at 4 °C in darkness, plants were transferred to long-day conditions (16 h light with 70 µmol photons m^–2^ s^−1^, 8 h dark) at 21–22 °C and 60% humidity for the times indicated.

### Cloning of constructs and generation of transgenic lines

To generate a reporter for trichoblasts, the previously described 437 bp promoter region from –386 to +48 of *EXPANSIN7* (*At1g12560*) (Cho and Cosgrove, 2002) was amplified using the forward primer 5′-ACGCGCGGCCGCGTGTTCAATTTAACTAATCATTG-3′ with a cleavage site for *Not*I and the reverse primer 5′-ACGCCTCGAGCTATTGAGAAGAATTTAAAGCT-3′ with an *Xho*I cleavage site, and ligated into pENTR1a DS to generate the *pEXP7:GUS* reporter. The construct was sequenced and recombined into pBGWFS7 by using the Gateway cloning system (Thermo Fisher Scientific, Waltham, MA, USA). Cloning of the *p35S:PSKR1-GFP* construct into pB7WG2.0 has been described previously (Hartmann *et al.*, 2013). Plant transformation and selection of transgenic plants was done as described (Kaufmann *et al.*, 2017).

### Preparation and analysis of cross-sections and GUS staining

*pEXP7:GUS*-expressing seedlings of the genetic backgrounds indicated were grown on plates for 5 d, collected, and stained with β-glucuronidase (GUS) staining solution (Vielle-Calzada *et al.*, 2000). Roots were separated from shoots and embedded in TechnoVit (Heraeus Kulzer, Wehrheim, Germany) as described in the manufacturer’s manual. Cross-sections of 10 µm thickness were prepared with a Leica RM 2255 microtome, collected on glass slides, embedded in CV Mount solution (Leica, Bensheim, Germany), and analyzed with an Olympus BX41 microscope. Pictures were taken with an Infinity 3S camera using the software Infinity Analyze 6.5 (Lumenera, Ottawa, Canada). The numbers of epidermal and cortical cells were counted on pictures of cross-sections. The cross-sectional area was determined with Fiji/ImageJ open-source software (https://imagej.net/Fiji) from the same pictures.

### RNA isolation and gene expression analyses

For microarray and quantitative reverse transcription–PCR (RT–qPCR) analyses, roots from 5-day-old seedlings that were grown on plates supplemented with or without 1 µM PSK under sterile condition were used. Total RNA was isolated with TRI-reagent (Sigma Aldrich, St. Louis, MO, USA) following the manufacturer’s instructions. RNA was dissolved in diethylpyrocarbonate (DEPC)-treated H_2_O and the quality and quantity of RNA was measured with a NanoDrop spectrometer (ThermoFisher Scientific). For RT-PCR and RT–qPCR, 1 µg of mRNA was digested with DNase I and subsequently reverse-transcribed with oligo(dT) primers. Quantitative PCR was performed with the Rotor-Gene SYBR Green PCR Kit (Qiagen, Venlo, The Netherlands) according to the manufacturer’s instructions. The reverse transcription products were amplified using gene-specific primers as indicated in Supplementary Table S1. Reactions were performed with a Rotor Gene Q cycler (Qiagen). Data (takeoff and efficiencies) were given by ‘Comparative quantification analysis’ from the cycler-corresponding Rotor Gene Q Series software (Qiagen). The fold change (FC) was calculated by normalization to the geometric mean of *ACT2* and *GAPC* expression. For statistical analysis, log_2_-transformed FC values were used. At least three independent biological replicates each with technical repeats were performed.

Microarray experiments were performed with three biological replicates of three samples each, the wild type, *tpst-1*, and *tpst-1* treated with 1 µM PSK. AraGene-1_0-st; Affymetrix microarray slides containing 38 408 transcripts were used for transcriptome analysis. Analysis of RNA quality, chip hybridization, and data processing were performed at the MicroArray Facility [Flanders Institute for Biotechnology (VIB), Leuven, Belgium]. Briefly, analysis was based on the Robust Multi-Array Average (RMA) expression values. To identify differentially expressed genes, the RMA expression values under the different conditions were compared with the LiMMA package of Bioconductor (Wettenhall and Smyth, 2004; Smyth, 2005). For each contrast of interest, we tested if it deviated significantly from 0 with a moderated *t*-statistic implemented in LiMMA. The resulting *P*-values were corrected for multiple testing with Benjamini–Hochberg correction to control the false discovery rate (Benjamini and Hochberg, 1995). All *P*-values given were corrected for multiple testing. A cut-off at a *P*-value of 0.001 was used to indicate differentially expressed genes combined with a cut-off at an FC of 2.

### Statistical analysis

Data sets were analyzed for normal distribution. In the case of normal distribution for all-pairwise comparison, an ANOVA with α=0.05 was run with Origin 8.5 software, whereas datasets that were not normally distributed were analyzed with a Kruskal–Wallis test with Bonferroni as the *P*-value adjustment method (α=0.05) by using the package ‘agricolae’ and statistics software R (https://CRAN.R-project.org/doc/FAQ/R-FAQ.html).

## Results

### Identification of genes differentially regulated by PSK

**Fig. 1.**
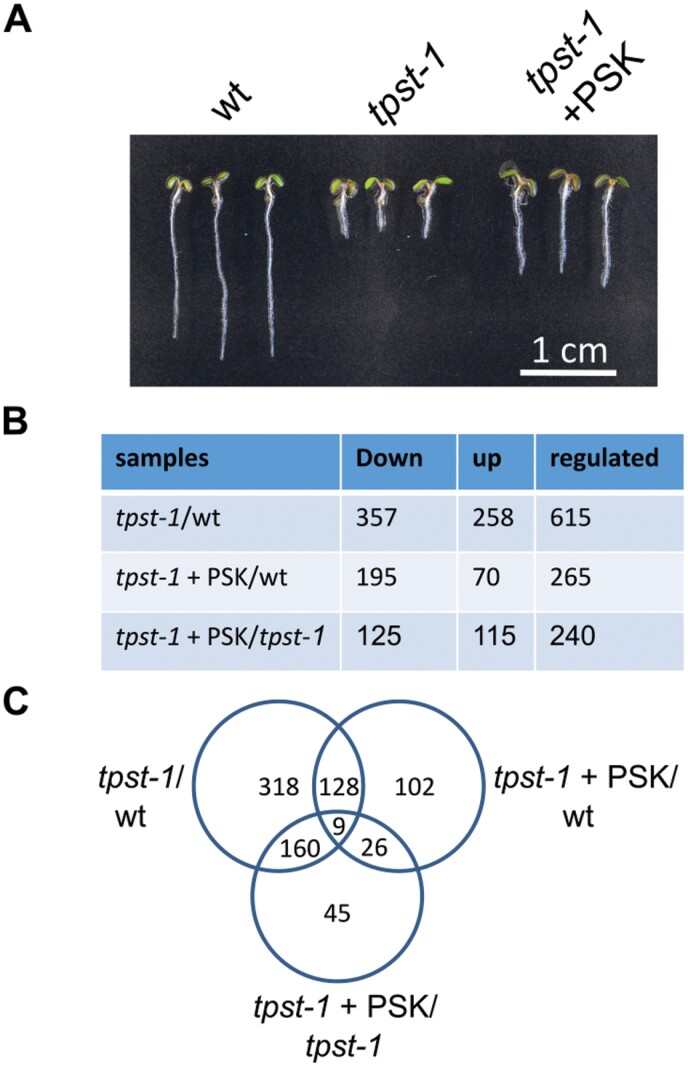
Transcriptomic analysis of genes regulated by sulfated peptides. (A) Representative seedlings of the wild type (wt), *tpst-1*, and *tpst-1* treated with 1 µM PSK grown for 5 d under long-day conditions. Complete roots were harvested and material was subjected to microarray analysis. Scale bar=1 cm. (B) Table with numbers of down- and up-regulated genes and the total number of genes regulated. The different samples compared are *tpst-1* versus the wt, *tpst-1*+PSK versus the wt, and *tpst-1*+PSK versus *tpst-1*. The microarray experiment was performed with three biological replicates each. A cut-off at a *P*-value of 0.001 was used to indicate differentially expressed genes combined with a cut-off at an FC of 2. (C) Venn diagram of genes regulated between the different genotypes and treatments. Total numbers of genes regulated are given.

**Fig. 2.**
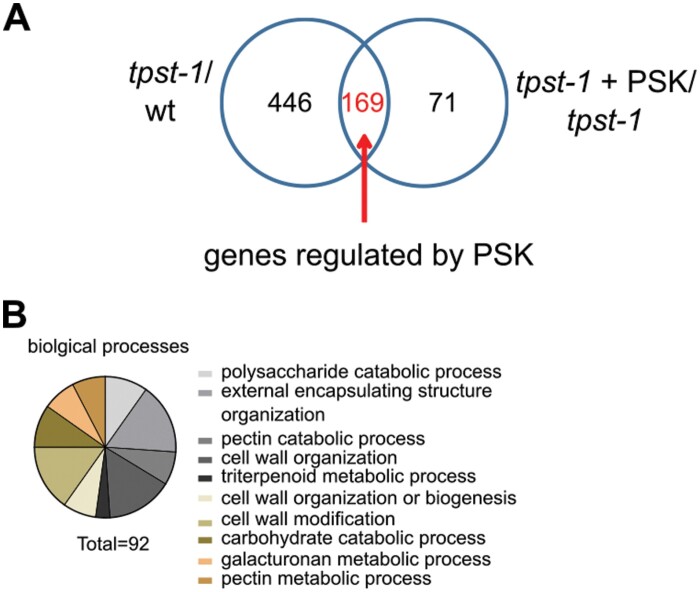
Identification of (over-represented) genes regulated by PSK. (A) Venn diagram that illustrates the number of genes regulated in the *tpst-1* versus the wild type (wt) and *tpst-1*+PSK versus *tpst-1*. The overlap between these samples identifies the number of genes that are regulated by PSK. (B) Biological processes that are over-represented among the genes that are regulated by PSK. The identified genes were analyzed by the PANTHER16.0 program (Mi *et al.*, 2019, 2021). A total of 92 genes could be assigned to specific, over-represented biological processes.

TPST is encoded by a single-copy gene in Arabidopsis. In *tpst* knockouts, production of sulfated peptides is abolished, leading to a sulfated ligand-free background. The *tpst-1* loss-of-function mutant of Arabidopsis has been a useful tool to study control of root development by sulfated peptide hormones (Komori *et al.*, 2009). Root growth induction by enhanced cell expansion is one of the best-characterized functions of the disulfated pentapeptide PSK (Matsubayashi *et al.*, 2006; Kutschmar *et al.*, 2009). In order to identify genes that are regulated by PSK and to compare these with genes that are regulated by other sulfated peptides, we performed a microarray experiment on roots of 5-day-old wild-type, *tpst-1*, and *tpst-1* seedlings that were treated with 1 µM PSK (Fig. 1A; for quantified data, see Fig. 4B). In total, we identified 615 genes that were differentially regulated between the wild type and the *tpst-1* mutant (FC ≥2; Fig. 1B). When we compared roots of *tpst-1* seedlings that were supplemented with PSK with roots of untreated *tpst-1* seedlings, 240 genes were found to be differentially regulated. The comparison between *tpst-1* seedlings treated with PSK and wild-type seedlings revealed 265 differentially expressed genes (Fig. 1B). The overlap of genes differentially regulated by PSK in *tpst-1* seedling roots and of genes differentially regulated between *tpst-1* and the wild type was determined to identify genes which are specifically regulated by PSK and not by other sulfated peptides (Fig. 2A). This comparison revealed 169 genes that are specifically regulated by PSK.

PSK regulates hypocotyl and root growth mainly by promoting cell expansion (Kutschmar *et al.*, 2009; Stührwohldt *et al.*, 2011). Cell expansion requires cell wall remodeling, which includes changes in cell wall composition and structure. To categorize the genes that are regulated by PSK, we analyzed the biological processes that are over-represented with the publicly available database PANTHER (Mi *et al.*, 2019, 2021). Here, we observed that PSK regulates genes over-represented in biological processes related to cell wall modification, organization, or biogenesis, which includes pectin and galacturonan metabolism as well as carbohydrate catabolism (Fig. 2B). Our data link gene expression changes induced by PSK signaling to growth triggered by PSK (Fig. 2A, B). We also detected an over-representation of genes linked to triterpenoid metabolism (Fig. 2B). The activity of one of the genes, *Marneral Synthase 1* (*MRN1*), has previously been linked to cell elongation (Go *et al.*, 2012).

**Fig. 3.**
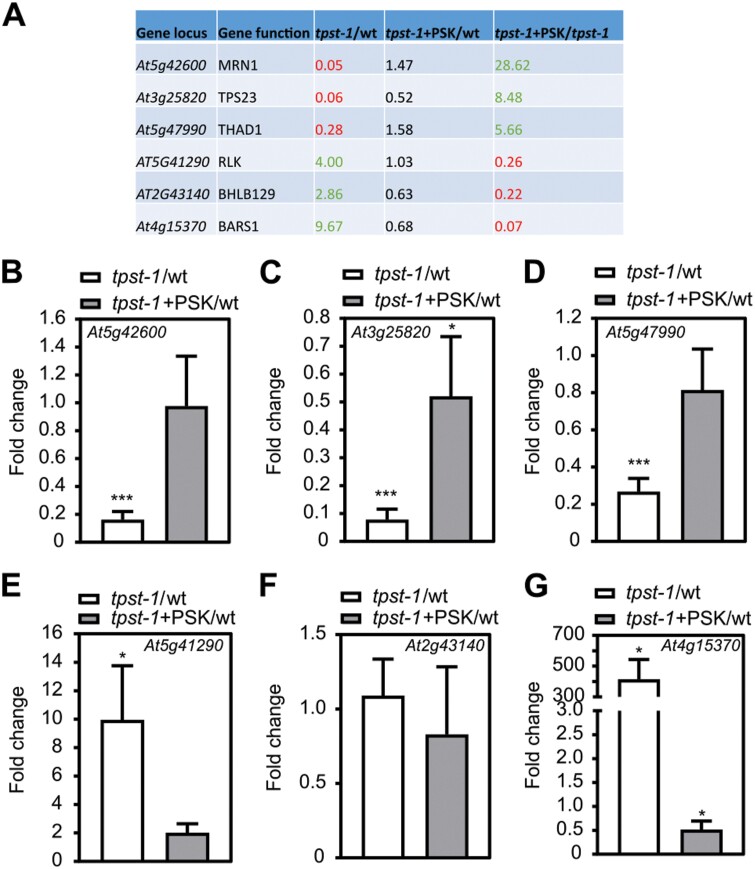
Verification of PSK-regulated genes in Arabidopsis roots by qPCR. (A) Table of chosen genes identified as PSK regulated within the microarray analysis. Six genes were chosen that were regulated by PSK, but not significantly regulated in *tpst-1*+PSK versus the wild type (wt). (B–G) Fold change of expression tested by qPCR of (B) *At5g42600* (*MRN1*), (C) *At3g25820* (*TPS23*), (D) *At5g47990* (*THAD1*), (E) *At5g41290* (a receptor-like kinase), (F) *At2g43140* (*BHLH129*), and (G) *At4g15370 (BARS1*). Roots were harvested from seedlings grown for 5 d under control conditions or treated with 1 µM PSK. qPCR was performed on three biological replicates with two technical repeats, and gene expression was normalized to two reference genes. Results are shown as fold changes in *tpst-1* versus the wt and *tpst-1*+PSK versus *tpst-1*. Each time point included pooled plant material of several independent seedlings. * and *** indicate significant differences compared with the control at *P*<0.05 or *P*<0.001, respectively (two-tailed *t*-test).

To independently verify differential gene expression in response to PSK, we selected six candidate genes and analyzed their relative transcript levels by RT–qPCR (Fig. 3). We tested three genes each that were down- or up-regulated by PSK (Fig. 3A). For *MRN1* (*At5g42600*), *Terpene Synthase-Like 23* (*TPS23*, *At3g25820*), and *Thalian-diol Desaturase* (*THAD1*, *At5g47990*) which encode terpene biosynthesis enzymes (Fig. 3A), we confirmed positive regulation by PSK (Fig. 3B–D). Of three genes that were negatively regulated by PSK according to the microarray experiment, a receptor-like kinase (*At5g41290*), a basic helix–loop–helix transcription factor (*BHLH129*, *At2g43140*), and *Baruol Synthase 1* (*BARS1*, *At4g15370*), we confirmed down-regulation by PSK for the receptor-like kinase and *BARS1* (Fig. 3E–G). To test for short- and long-term regulation of genes by PSK, we exposed *tpst-1* seedlings in hydroponic culture to 100 nM PSK for 4, 8, 12, 16, 20, 24, and 48 h or kept them as controls without PSK (Supplementary Fig. S1). Genes of baruol, marneral, and thalianol synthesis are organized in clusters, and genes within a cluster were coordinately regulated (Field and Osbourn, 2008). For the three baruol biosynthesis genes *BARS1*, *CYP705A3*, and *CYP705A2* identified in the microarray, we observed significant down-regulation of transcripts over time, with the highest reduction in expression after 48 h (Supplementary Fig. S1). Expression of the three marneral biosynthesis genes and of the three thalianol genes increased over time in response to PSK, suggesting that the microarray data reliably revealed genes differentially regulated by PSK (Supplementary Fig. S1).

### Growth and lateral root development is impaired more in the *tpst-1 pskr1-3 pskr2-1* triple mutant than in *tpst-1*

Genes that were regulated similarly in *tpst-1* versus the wild type and in *tpst-1* treated with PSK versus the wild type were defined as regulated by sulfated peptides other than PSK. Here, we identified 128 genes (Supplementary Fig. S2A). Putative candidate peptides responsible for the regulation of these genes are PSYs, RGFs, and CIFs. The biological processes that were over-represented in this group included responses to reactive oxygen species (ROS), reactive nitrogen species, salicylic acid, and metal and iron ions (Supplementary Fig. S2B) in agreement with recently published data that showed control of root meristem size by RGF1 through ROS signaling (Yamada *et al.*, 2020). Overall, genes over-represented in the *tpst-1* mutant could be assigned to cell wall modifications, ROS, nitric oxide, and secondary metabolism (Supplementary Fig. S3).

**Fig. 4.**
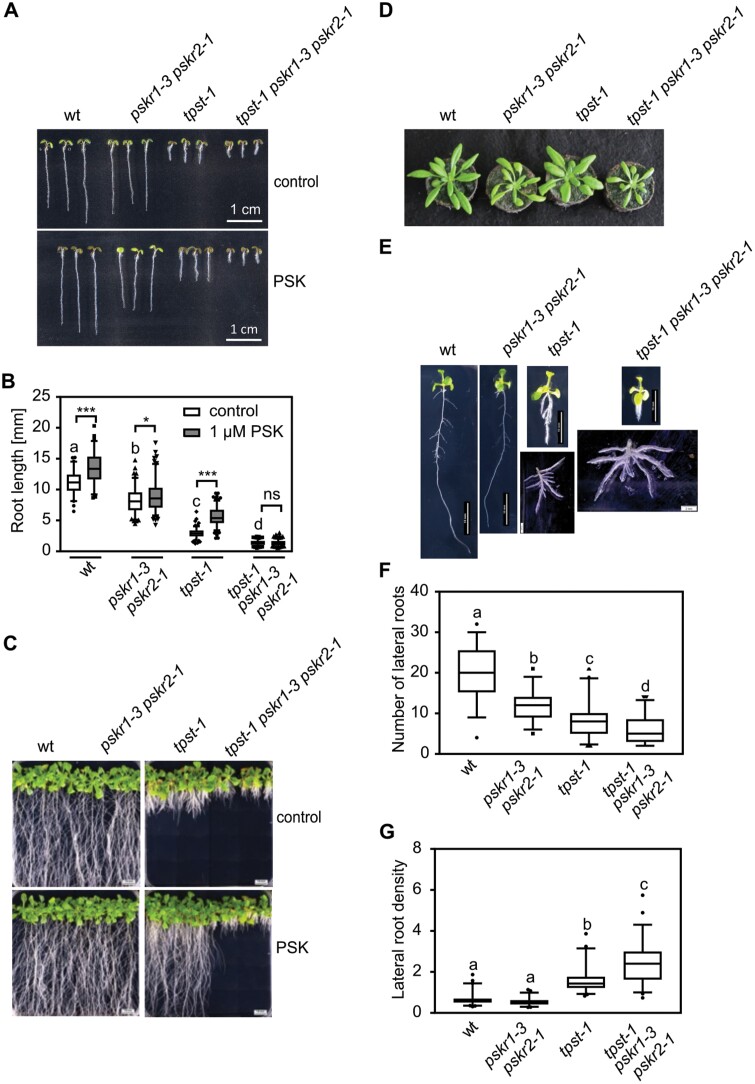
A *tpst-1 pskr1-3 pskr2-1* triple mutant shows an unexpected, synergistic phenotype. (A) Representative seedlings of the wild type (wt), *pskr1-3 pskr2-1*, *tpst-1*, and *tpst-1 pskr1-3 pskr2-1* grown with or without 1 µM PSK for 5 d under long-day conditions. Scale bar=1 cm. (B) Root length (mm) of the 5-day-old wt, *pskr1-3 pskr2-1*, *tpst-1*, and *tpst-1 pskr1-3 pskr2-1* treated without or with 1 µM PSK. (C, D) Representative images of the respective genotypes grown (C) for 3 weeks under sterile conditions with and without PSK or (D) for 4 weeks on soil under long-day conditions. (E) Representative images of plants grown for 11 d under sterile growth conditions. For wt, *tpst-1*, *pskr1-3 pskr2-1*, and *tpst-1 pskr1-3 pskr2-1*, a representative example of lateral root development is shown. Scale bars represent the indicated lengths. (F) Number of lateral roots and (G) lateral root density of 11-day old plants of the wt, *pskr1-3 pskr2-1*, *tpst-1*, and *tpst-1 pskr1-3 pskr2-1*. The root and the shoot were separated and lateral roots were spread to determine initiation sites of lateral roots. Experiments were performed at least three times with similar results. Data are shown for one representative experiment as the mean ±SE, (B) *n*≥68, (F) *n*≥46, (G) *n*≥46. Different letters indicate significant differences (Kruskal–Wallis, *P*<0.05). In (B), controls were compared by Kruskal–Wallis (*P*<0.05) and significance between control and PSK treatment was tested by a two-tailed *t*-test. *** and * indicate significant differences at *P*<0.001 or *P*<0.05, respectively.

Tyrosine sulfation is a prerequisite for activity of sulfated peptides. Consequently, loss of peptide signaling in the *tpst-1* mutant is complemented by the addition of sulfated peptides (Komori *et al.*, 2009; Matsuzaki *et al.*, 2010; Doblas *et al.*, 2017). The PSK receptor double knockout mutant *pskr1-3 pskr2-1* is insensitive to PSK, for instance with regard to root growth promotion (Kutschmar *et al.*, 2009) (Fig. 4A, B). The analysis of the gene expression data revealed regulation of genes that we could not clearly categorize as being regulated by PSK or other sulfated peptides (see examples in Supplementary Fig. S4). As well as genes that were regulated in *tpst-1*, but were not regulated by PSK (category A), we identified genes that were regulated in *tpst-1* with only partial restoration of expression by PSK (category B) and genes that were only differentially expressed when comparing *tpst-1* supplemented with PSK with the wild type (category C) (Supplementary Fig. S4), suggesting crosstalk between TPST-dependent signaling pathways. To be able to more clearly address PSK-dependent root developmental processes in the sulfated peptide-deficient background, we created a triple mutant by crossing the *tpst-1* mutant with the PSKR double mutant, *pskr1-3 pskr2-1*. Knockout of all three genes was confirmed by RT-PCR (Supplementary Fig. S5A). We expected that the *tpst-1 pskr1-3 pskr2-1* triple mutant should phenocopy the *tpst-1* mutant but should be insensitive to PSK as is the *pskr1-3 pskr2-1* mutant. Unexpectedly, the combined knockout of *PSKR* and *TPST* genes had synergistic effects (Fig. 4A, B). The *tpst-1 pskr1-3 pskr2-1* and *pskr1-3 pskr2-1* seedlings were insensitive to PSK, as expected (Fig. 4A, B). However, whereas the primary roots of *pskr1-3 pskr2-1* and *tpst-1* seedlings were reduced in length by 25.5% and 73.5%, respectively, *tpst-1 pskr1-3 pskr2-1* triple mutant roots were reduced by 87.4% which is significantly more than in *tpst-1* (Fig. 4A, B). Synergistic effects in the *tpst-1 pskr1-3 pskr2-1* triple mutant compared with *tpst-1* and *pskr1-3 pskr2-1* were also observed with regard to overall plant architecture and rosette size (Fig. 4C, D).

The observation that several genes related to lateral root development were regulated in the *tpst-1* mutant (Supplementary Fig. S6) prompted us to analyze lateral roots in the *tpst-1*, *pskr1-3 pskr2-1*, and *tpst-1 pskr1-3 pskr2-1* mutants. Lateral root density in *pskr1-3 pskr2-1* seedlings was comparable with that in the wild type, whereas the number of lateral roots was significantly reduced, probably due to a shorter primary root (Fig. 4E–G) (Kutschmar *et al.*, 2009). The *tpst-1* mutant had a significantly reduced number of lateral roots compared with the wild type and *pskr1-3 pskr2-1*, whereas lateral root density was increased, revealing that sulfated peptides other than PSK control lateral root density. In *tpst-1 pskr1-3 pskr2-1* seedlings, these phenotypes were even more pronounced, with fewer lateral roots and a higher lateral root density than in *tpst-1* seedlings (Fig. 4F, G). These findings raised the hypothesis that lateral root development could, to some extent, be triggered by PSKR activity independent of the sulfated ligand PSK. Likewise, it is conceivable that residual receptor activity exists in *pskr1-3 pskr2-1*, and signaling via this residual receptor activity is saturated by endogenous PSK in accord with the observation that the *pskr1-3 pskr2-1* mutant is insensitive to exogenous PSK (Kutschmar *et al.*, 2009). Also, residual peptide sulfation activity cannot be completely excluded in *tpst-1*. However, *tpst-1* shows a 30-fold higher sensitivity toward exogenous PSK compared with the wild type, indicating that PSKRs are largely in an unbound state (Stührwohldt *et al.*, 2011). In this case, expressing additional receptors should not promote a PSK response.

**Fig. 5.**
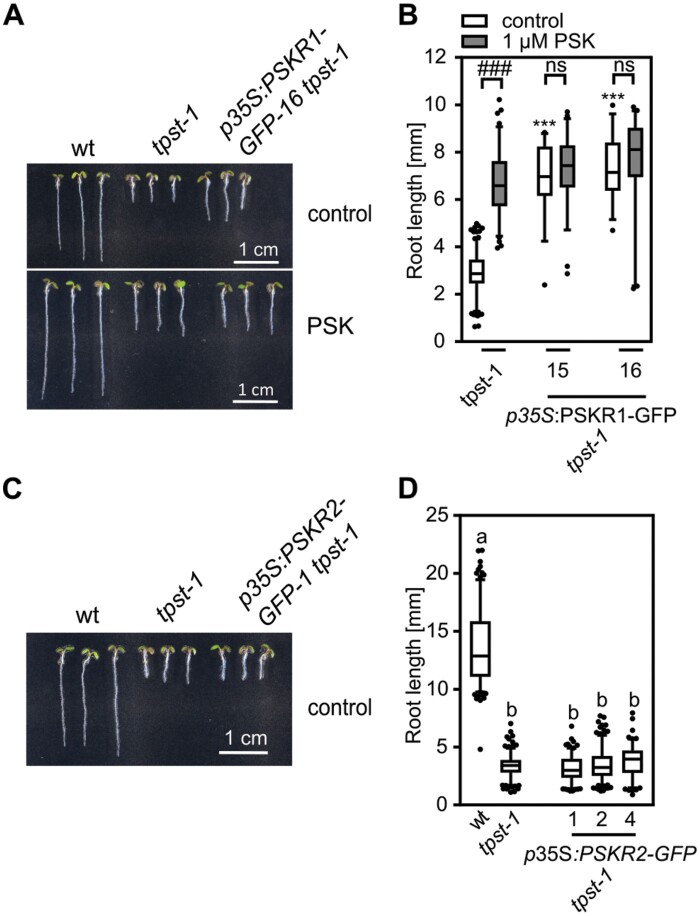
Overexpression of *PSKR1* in the *tpst-1* background promotes root growth. (A, C) Representative seedlings of the wild type (wt), *tpst-1*, *p35S*:PSKR1-GFP *tpst-1*, and *p35S*:PSKR2-GFP *tpst-1* grown for 5 d without (A, C) and with 1 µM PSK (A). (B, D) Root lengths (mm) of (B) the wt, *tpst-1*, and two independent lines of *p35S*:PSKR1-GFP *tpst-1*, and (D) the wt, *tpst-1*, and *p35S*:PSKR1-GFP *tpst-1* supplemented without (B, D) or with 1 µM PSK (B). Numbers indicate independent transgenic lines. (B) Experiments were performed at least three times with similar results. Data are shown for one representative experiment as the mean ±SE. (D) The experiment was performed once. Data are shown as the mean ±SE. (B) *n*≥31, (D) *n*≥122. In (B), *** indicates significant differences from the wt control at *P*<0.001 (two-tailed *t-*test). ^###^ indicates a significant difference in comparison with the untreated control at *P*<0.001 (two-tailed *t*-test). In (D), different letters indicate significant differences (Kruskal–Wallis, *P*<0.05). In (A) and (C), scale bars represent 1 cm.

To address this issue, we overexpressed *PSKR1* or *PSKR2* in the *tpst-1* mutant background (Fig. 5; Supplementary Fig. S5B, C) under the control of the constitutive 35S promoter. Overexpression of *PSKR1* or *PSKR2* was verified by semi-quantitative RT-PCR for two and three lines, respectively (Supplementary Fig. S5B, C), and the lines were analyzed for primary root elongation as an easy-to-monitor readout. Strikingly, overexpression of *PSKR1* in the *tpst-1* background induced root growth (Fig. 5A, B) while *PSKR2* overexpression did not (Fig. 5C, D), suggesting that PSKR1 has growth-promoting activity in the absence of sulfated ligands. Root elongation of the PSKR1 overexpressors in the *tpst-1* background was comparable with that of *tpst-1* seedlings treated with PSK and was not further promoted by addition of PSK (Fig. 5A, B). These findings indicated that lack of sulfated PSK can be compensated for by increasing the abundance of PSKR1; lines with high PSKR1 abundance have saturated PSKR1 signaling independent from its ligand (Fig. 5A, B).

### PSKRs signal non-hair cell fate through WER expression

**Fig. 6.**
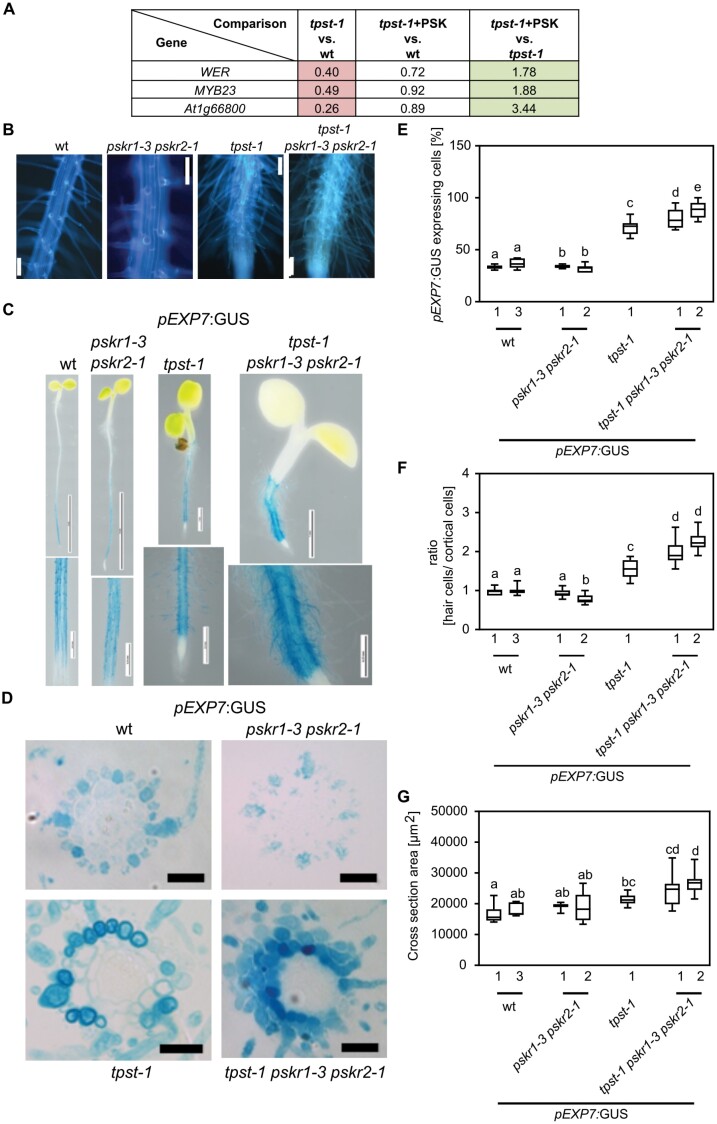
TPST and PSKRs control root hair formation by altering position-dependent epidermal cell fate determination. (A) Table with fold change of *WER*, *MYB23*, and *At1g66800* identified from the microarray experiment. Microarray experiments were performed with three biological replicates. (B) Autofluorescence imaging of the root hair zone of the wild type (wt), *pskr1-3 pskr2-1*, *tpst-1*, and *tpst-1 pskr1-3 pskr2-1*. Scale bar=100 µm. (C) Representative images of 5-day-old wt, *pskr1-3 pskr2-1*, *tpst-1*, and *tpst-1 pskr1-3 pskr2-1* seedlings expressing *pEXP7*:*GUS*. Scale bars represent the indicated lengths. (D) Representative cross-sections of 5-day-old *pEXP7:GUS* seedlings; scale bar=50 µm. (E) Quantification of cells expressing *pEXP7:GUS* as a marker for trichoblasts. (F) Ratio of *pEXP7:GUS*-expressing hair cells to cortical cells in the genotypes indicated. (G) Cross-sectional area (µm^2^) determined from cross-sections of *pEXP7p*:*GUS*-expressing seedlings. Numbers indicate independent transgenic lines. Experiments were performed at least three times with similar results. Data are shown for one representative experiment as the mean ±SE, (E) *n*≥9, (F) *n*≥9, (G) *n*≥9. Different letters indicate significant differences (Kruskal–Wallis, *P*<0.05).

We found that the *hypersensitive to Pi starvation 7* (*hps7*) mutant, that is allelic to *tpst-1*, shows a root hair phenotype that has, however, not been addressed previously (Kang *et al.*, 2014). Furthermore, genes involved in the control of root hair formation are differentially regulated by PSK based on our microarray data (Fig. 6A). We therefore asked whether TPST and PSKRs play a similar synergistic role in root hair formation to that in root growth and lateral root formation. *tpst-1* displays an abnormal root hair phenotype (Fig. 6B). Root hair formation in the *tpst-1 pskr1-3 pskr2-1* triple mutant was even more pronounced (Fig. 6B). To analyze root hair repression by the TPST–PSKR signal pathway in more detail, we transformed wild-type, *tpst-1*, *pskr1-3 pskr2-1*, and *tpst-1 pskr1-3 pskr2-1* plants with the trichoblast-specific reporter *pEXP7:GUS* (Cho and Cosgrove, 2002) to visualize and quantify root hairs (Fig. 6C). Promoter activity excluding the meristematic zones was detected in roots of all genotypes (Fig. 6C).

To determine the percentage of *pEXP7:GUS-*expressing cells, the ratio of hair cells to cortical cells and the cross-sectional areas of the mutant roots, we made cross-sections from the root hair zone (Fig. 6D). In two *pEXP7:GUS pskr1-3 pskr2-1* lines, the percentage of cells with root hair identity was 34.1% and 32.4%, respectively, compared with 33.5% and 36.8% in two *pEXP7:GUS* wild-type lines (Fig. 6E). In *pEXP7:GUS tpst-1* seedlings, 71.9% of epidermal cells had root hair cell identity, indicating that sulfated peptide signaling determines non-hair cell fate (Fig. 6E). In *pEXP7:GUS tpst-1 pskr1-3 pskr2-1* seedlings, the number of root hair cells was significantly higher than in *pEXP7:GUS tpst-1* seedlings, with 79.5% and 88.4% of epidermal cells expressing the hair cell marker (Fig. 6E), indicating that root hair formation was suppressed by sulfated peptide signaling, in part via PSKRs.

**Fig. 7.**
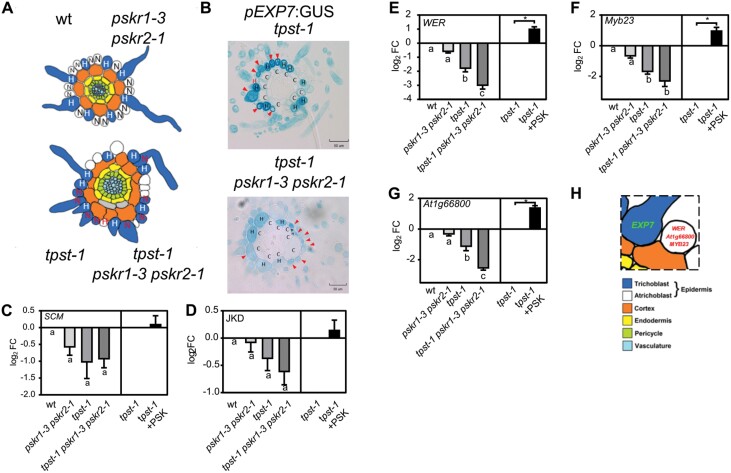
TPST and PSKRs control root hair formation by regulation of three marker genes for non-hair cell fate. (A) Schematic presentation indicating the position of trichoblasts and cortical cells. Trichoblasts are in an H (root hair) position in the wild type (wt) and *pskr1-3 pskr2-1*. In *tpst-1* and *tpst-1 pskr1-3 pskr2-1*, they are developed in H and N (non-hair root) positions. (B) Representative cross-sections of 5-day-old *tpst-1* and *tpst-1 pskr1-3 pskr2-1* seedlings expressing *pEXP7:GUS*. Arrows indicate *pEXP7:GUS*-expressing cells in the N position.The red H marks a cell that is in an H position, but does not express *pEXP7:GUS.* Scale bars=50 µm (C–G) Log_2_ fold change of expression tested by qPCR of non-hair cell fate marker genes (C) SCM (LRR receptor-like kinase Scrambled), (D) JKD (Jackdaw), (E) WER (Werewolf), (F) Myb23, and (G) At1g66800. Seedlings were grown for 5 d under control conditions or treated with 1 µM PSK and subjected to RT–qPCR analysis. RT–qPCR was performed on three biological replicates with two technical repeats, and gene expression was normalized to two reference genes and is shown as log_2_ fold change. Each time point included pooled plant material of several independent seedlings. Asterisks indicate significant differences between *tpst-1* and *tpst-1* treated with PSK. Different letters indicate significant differences (Kruskal–Wallis, *P*<0.05). (H) Schematic presentation indicating expression of marker genes *EXP7*, *WER*, *MYB23*, and *At1g66800* in Arabidopsis wt seedlings.

Hair cell formation in Arabidopsis is determined by the position of epidermal cells with regard to the cortical cell layer (Ma *et al.*, 2001; Salazar-Henao *et al.*, 2016). Hair cells touch two cortex cells while non-hair cells border on a single cortex cell (Fig. 7A). To gain more insight into the activity of TPST and PSKRs in the position-dependent control of root hair formation, we analyzed hair and cortical cells in more detail. While the root cross-sectional area increased significantly in *tpst-1* and *tpst-1 pskr1-3 pskr2-1* compared with the wild type (Fig. 6G), the number of epidermal cells remained constant in all four genotypes (Supplementary Fig. S7A). Further, the number of cortex cells only increased slightly in the mutants (Supplementary Fig. S7B). Consequently, we determined the ratio of hair cells to cortex cells that was 1:1 in the wild type and *pskr1-3 pskr2-1* (Fig. 6F). The ratio of hair to cortex cells increased to 1.5:1 in *tpst-1* and to 2.1:1 in *tpst-1 pskr1-3 pskr2-1*. The increase in root hair cells in *tpst-1* and *tpst-1 pskr1-3 pskr2-1* was not a result of more hair positions above two cortical cells, but rather due to loss of position-dependent determination of epidermal cell fate with trichoblasts developing at non-hair positions, as illustrated in Fig. 7A and experimentally shown for *tpst-1* and *tpst-1 pskr1-3 pskr2-1* in Fig. 7B.

To identify the downstream targets of the TPST/PSKR1 signals that determine non-hair cell fate, we tested expression of two major regulators of non-hair cell fate, the transcription factor JACKDAW (JKD) (Hassan *et al.*, 2010), expressed in cortical cells, and the LRR-RLK SCRAMBLED (SCM) localized at the plasma membrane of trichoblasts. Expression of either gene was not significantly different between wild-type, *pskr1-3 pskr2-1*, *tpst-1*, or *tpst-1 pskr1-3 pskr2-1* roots (Fig. 7C, D). Further, PSK did not induce the expression of *JKD* or *SCM*, indicating that they are not transcriptional targets of TPST/PSKR signaling (Fig. 7C, D). Next, we tested expression of the downstream atrichoblast-specific transcription factor genes *WER*, *MYB23*, and *At1g66800*. In our microarray experiment, all three transcription factors were down-regulated in the *tpst-1* mutant compared with the wild type, and induced by PSK, for *WER* and *MYB23* by an FC of 1.66 and 1.78, respectively (Fig. 6A). Expression analysis by RT–qPCR showed that all three transcription factors were slightly, but not significantly, reduced in the *pskr1-3 pskr2-1* mutant (Fig. 7E–G). However, all three genes were significantly down-regulated in *tpst-1* versus the wild type and further repressed in *tpst-1 pskr1-3 pskr2-1* compared with *tpst-1* (Fig. 7E–G), again revealing a synergistic effect of the mutations. Expression of *WER*, *MYB23*, and *At1g66800* was induced by PSK in *tpst-1*, indicating that PSKR signaling maintains non-hair cell fate, with PSK enhancing the signal output (Fig. 7E–G). In conclusion, PSK acts downstream or independent of JKD and SCM, and promotes expression of *WER*, *MYB23*, and *At1g66800*. In this pathway, PSKR may act dependent on and independent of its ligand PSK to promote non-hair cell fate (Fig. 7H).

## Discussion

### Transcriptional crosstalk between peptide signaling pathways

The use of the *tpst-1* mutant for our transcriptome analysis turned out to be a helpful approach to identify genes that are regulated by PSK in roots. A time course expression analysis of triterpenoid synthesis genes showed that gene regulation detected in steady-state expression profiles by microarray analysis in many cases also successfully predicted gene regulation in the short term (Fig. 3; Supplementary Fig. S1). Growth of above-ground parts of plants overexpressing thalianol synthase (THAS) or MRN1 was significantly inhibited, indicating that an excess amount of thalianol, marnerol, or their derivatives might be detrimental for shoot growth. On the other hand, plants overexpressing THAS have longer roots than the wild type or *thas* knockout lines, suggesting a root-specific growth-promoting effect (Field and Osbourn, 2008; Field *et al.*, 2011).

The *tpst-1* mutant, when compared with the wild type, revealed genes regulated by sulfated peptides in general. For some genes, regulation by several sulfated peptides seems likely. Some of the genes that were identified as regulated in *tpst-1* were only partially regulated by PSK, indicating crosstalk between different sulfated peptide signaling pathways (Zhou *et al.*, 2010; Whitford *et al.*, 2012; Zhang *et al.*, 2018). Common gene targets and crosstalk between different signaling peptide pathways agree with the observation that root growth, which is stunted in *tpst-1*, is fully restored by the joint application of PSK, PSY1, and RGF1 (Matsuzaki *et al.*, 2010). Joint application of PSK and PSY1 restores cell elongation activity, but does not affect meristematic activity in *tpst-1*, as evidenced by meristem size (Matsuzaki *et al.*, 2010). Single peptides partially rescue the short-root phenotype, whereas PSK and RGF1 together promote root growth in a non-additive manner (Matsuzaki *et al.*, 2010), indicating crosstalk between the signaling pathways. The transcriptome approach taken here provides a repertoire of data and allows the assignment of genes that are regulated by PSK, by sulfated peptides other than PSK, or genes that are regulated by PSK and one or more additional sulfated peptides.

### Downstream targets of PSK and PSK receptor signaling in root growth

*TPST* is highly expressed in lateral root primordia (Komori *et al.*, 2009), is induced by auxin, and affects the expression of *PLT1/2*, *PIN*, and auxin biosynthetic genes (Zhou *et al.*, 2010). Since lateral roots are induced by auxin (Péret *et al.*, 2009), these findings agree with a role for TPST in the control of lateral root density (Fig. 4). Overexpression of several Golven (RGF, CLEL) peptides in Arabidopsis reduced lateral root density (Fernandez *et al.*, 2013), indicating that Golven peptides regulate lateral root architecture jointly with other sulfated peptides, such as PSK or PSY1, possibly at different developmental stages.

In addition to primary root elongation and lateral root formation, root system architecture is regulated by tyrosine-sulfated peptides through the control of root hair development. Sulfated peptides Golven 4 (RGF7, CLEL4) and Golven 8 (CLEL5) have been implicated in root hair elongation, as indicated by shorter root hairs in lines where peptides were silenced or knocked out (Fernandez *et al.*, 2013). In *tpst-1*, more root hairs develop, indicating that sulfated signaling peptides act to maintain non-hair cell fate (Fig. 6). In addition to the *tpst-1* T-DNA insertion line (Komori *et al.*, 2009), other *TPST* mutants have been identified, namely the active quiescent center mutants (*aqc1-1* to *aqc1-3*) (Zhou *et al.*, 2010) and the *hypersensitive to Pi starvation 7* (*hps7*) mutant (Kang *et al.*, 2014). The *hps7* mutant displayed a similar root hair phenotype to *tpst-1* that was exaggerated by phosphate starvation, with root hair formation extending toward the tip close to the meristem, revealing a link between TPST-dependent signaling in nutrient stress adaptation. Support for a broader contribution of sulfated peptide signaling in abiotic stress responses comes from recent reports on the role of PSK in mediating osmotic stress tolerance (Stührwohldt *et al.*, 2021).

In root hair development, TPST-dependent signals appear to act downstream of the transcription factor JKD and the kinase SCM. Both participate in the cortex to epidermis cell communication, and ensure that root hairs form from epidermal cells that are in contact with two cortex cells, but not from epidermal cells that are in touch with a single cortex cell, leading to natural spacing of root hair cell files in Arabidopsis (Hassan *et al.*, 2010). JKD and SCM act upstream of the transcription factors WER and MYB23 that are expressed atrichoblast specifically (Schiefelbein *et al.*, 2014). SCM represses *WER* transcripts, preferentially in hair cells. WER and MYB23 are key repressors of root hair identity in epidermal cells that, when knocked out, lead to a hairy root phenotype (Matsui *et al.*, 2005; Grierson *et al.*, 2014). Strikingly, atrichoblast-specific expression of PSKR1 is sufficient to promote root growth (Hartmann *et al.*, 2013), providing an unexpected spatial link between PSKR activity, promotion of root elongation, and inhibition of root hair formation. The involvement of PSKR1 signaling in root hair development is manifested by the reinforced physiological and transcriptional effects in the *tpst-1 pskr1-3 pskr2-1* triple mutant.

While expression of *JKD* and *SCM* was not altered in *tpst-1*, transcript levels of *WER* and *MYB23* were reduced compared with the wild type and were at least partially restored by PSK (Fig. 7), suggestive of a role for sulfated peptide signaling in suppression of trichoblast differentiation. In *tpst-1* seedlings, the control of hair cell identity by cortex cells is lost and root hairs develop at non-hair positions. Together, these findings indicate that protein sulfation by TPST is a crucial element in cell–cell communication that helps to establish cell identities in the root epidermis.

### Ligand-(in)dependent regulation of receptor activities

Hormone receptors are considered to be activated through binding of their respective ligand (Hohmann *et al.*, 2017). High-affinity binding of PSK to the ectodomain of its receptor depends on Tyr sulfation of the PSK proprotein by TPST (Wang *et al.*, 2015). PSK binding to PSKR1 at the island domain within the ectodomain promotes binding to the co-receptor BAK1/SERK3 or other members of the SERK family (Ladwig *et al.*, 2015; Wang *et al.*, 2015; Hohmann *et al.*, 2017). Yet, some binding of the ectodomains of PSKR1 and its co-receptor was observed in the absence of PSK (Wang *et al.*, 2015). Whether the ligand-free PSKR/BAK1 heterodimerization results in receptor activation has not been explored. Mutual phosphorylations between the receptor and co-receptor activate the cytosolic PSKR kinase and initiate signaling. The soluble kinase domain of PSKR1 displays auto- and transphosphorylation activities (Hartmann *et al.*, 2015; Kaufmann and Sauter, 2017). Among other residues, two Ser residues in the juxtamembrane region are autophosphorylated by the soluble PSKR1 kinase. This phosphorylation not only occurs *in vitro*, but was demonstrated to also occur *in planta* on the full-length receptor (Kaufmann and Sauter, 2017). Site-directed mutagenesis of the phosphorylated Ser residues altered substrate phosphorylation activity *in vitro* and shoot growth *in planta*, indicating that the soluble kinase domain of PSKR1 acquires an active conformation. Root growth promotion by overexpression of PSKR1 in the PSK-deficient *tpst-1* background suggests that the receptor can similarly acquire an active state in the absence of a sulfated ligand by either binding to an unsulfated ligand, receptor modification, or interaction with other proteins. It is conceivable that unsulfated PSK is released into the apoplast in the *tpst-1* mutant that binds to and activates PSKR1. *In vitro* binding of the unsulfated peptide to the LRR of PSKR1 occurs with a 30-fold lower affinity than binding of PSK (Wang *et al.*, 2015). Similarly, supply of unsulfated PSK to seedlings promotes root growth, yet with a 1000-fold lower activity than PSK (Kutschmar *et al.*, 2009). Given a high enough concentration, unsulfated ligand may contribute to basal receptor activity in *tpst-1*.

PSKR1 and related LRR-RLKs such as BRI1 interact in multimeric protein complexes, the composition of which may differ depending on cell type, physiological state, and environmental signals. No studies have yet been done to evaluate the impact of the protein composition of the PSKR module on receptor output, but it is conceivable that the phosphorylation status and protein interactions impact the conformation and activity of the intracellular kinase. Analysis of the *tpst-1 pskr1-3 pskr2-1* mutant revealed a strong synergistic effect of ligand and receptor knockout in all three root phenotypes analyzed, namely primary root elongation, lateral root formation, and root hair formation, that may be explained by ligand-independent receptor activation or by receptor activation through unsulfated ligand.

## Supplementary data

The following supplementary data are available at *JXB* online.

Fig. S1. Expression analysis of genes involved in triterpene synthesis in response to PSK treatment.

Fig. S2. Genes regulated by sulfated peptides other than PSK.

Fig. S3. Regulated genes over-represented in the *tpst-1* mutant.

Fig. S4. Genes that could not be clearly categorized as regulated by PSK or other sulfated peptides and were sorted by different categories.

Fig. S5. Semi-quantitative RT-PCR analysis.

Fig. S6. Differentially regulated genes with functions in lateral root growth and development.

Fig. S7. Analysis of epidermal and cortical cell numbers.

Table S1. Primers used in this study.

Table S2. Differentially expressed genes identified by microarray analysis.

erab233_suppl_Supplementary_File001

erab233_suppl_Supplementary_File002

## Data Availability

The data supporting the findings of this study are available from the corresponding author, Margret Sauter, upon request.
