# Supplementary material for: Tyrosylprotein sulfotransferase-dependent and -independent regulation of root development and signaling by PSK LRR receptor kinases in Arabidopsis
Source: J Exp Bot. 2021 May 24;72(15):5508–21. doi: 10.1093/jxb/erab233 (PMC8318253; doi:10.1093/jxb/erab233)
Supplement: erab233_suppl_Supplementary_File002 [file erab233_suppl_supplementary_file002.pdf]

Supplementary Figure S1

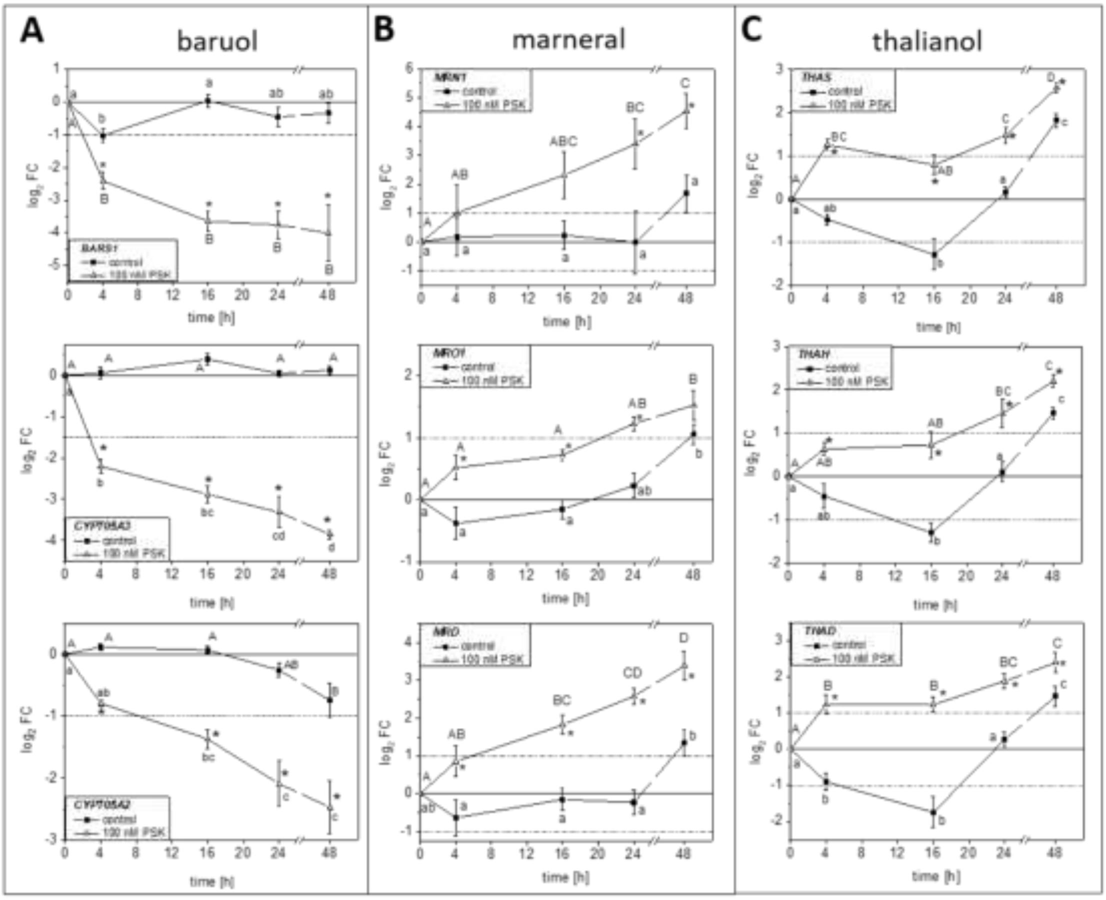

Supplementary Figure S1: Expression analysis of genes involved in triterpene synthesis in response to PSK treatment. $\log_2$ fold chance of expression of **(A)** baruol, **(B)** marneral and **(C)** thalianol biosynthesis genes. Expression was tested in roots of *tpst-1* seedlings that were grown for five days in hydroponic culture and treated with or without 100 nM PSK for the time indicated. Small or capital letters indicate significant differences between time points of control samples or PSK-treated samples (ANOVA, post-hoc Bonferroni, $p<0.05$), whereas asterisks indicate significant difference between treatments at a specific time point (Student t-test; $p<0.05$; n=3 three).

Supplementary Figure S2

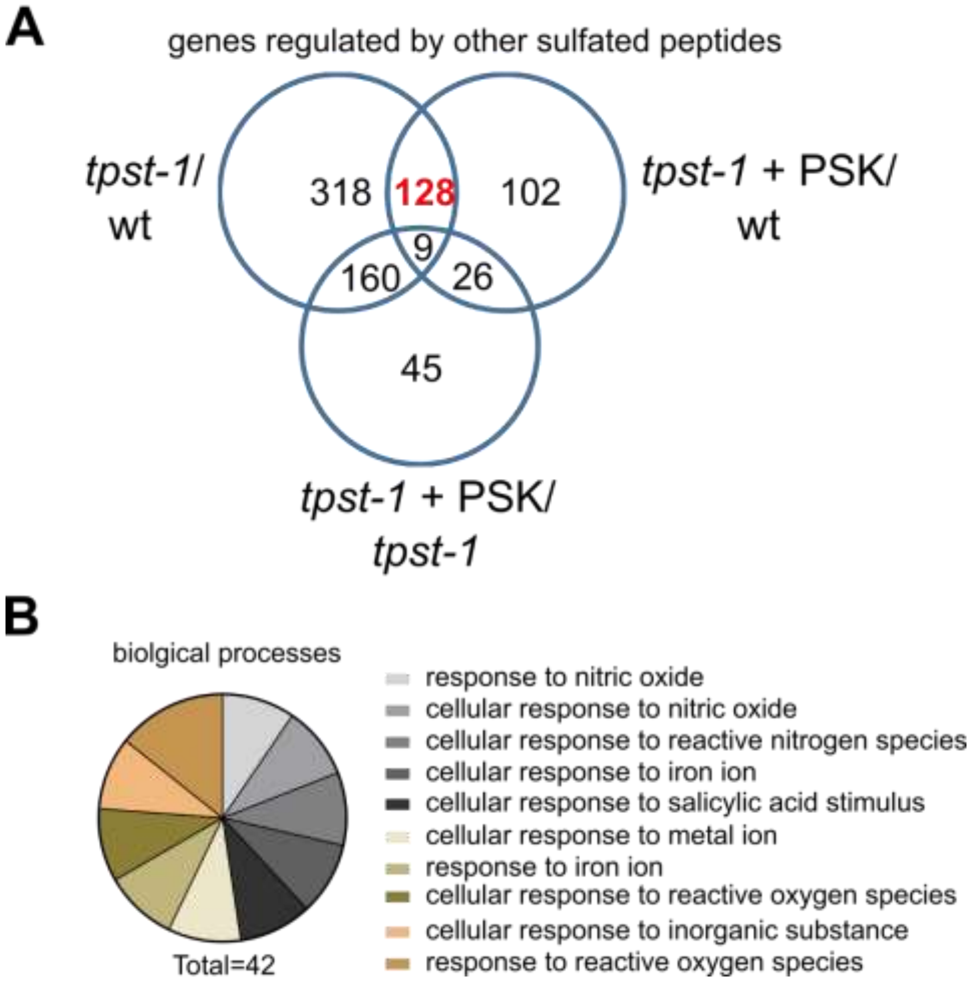

Supplementary Figure S2: Genes regulated by other sulfated peptides than PSK. **(A)** Venn diagram of genes regulated between the different genotypes and treatments indicated. Total number of genes regulated are given. The red number indicates the number of genes regulated by other sulfated peptides than PSK **(B)** Biological processes that are overrepresented among the genes that are regulated by other sulfated peptides than PSK. The identified genes were analyzed by the PANTHER16.0 programme (Mi *et al.*, 2019; Mi *et al.*, 2021). A total of 42 genes could be assigned to biological processes that are overrepresented.

Supplementary Figure S3

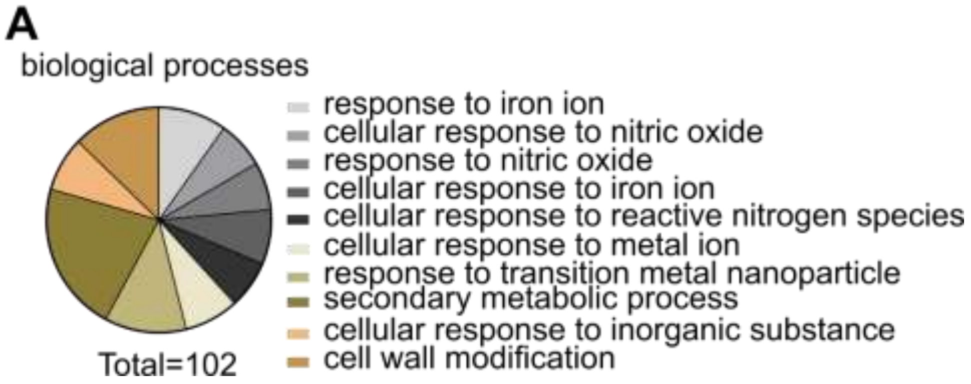

Supplementary Figure S3: Regulated genes overrepresented in the *tpst-1* mutant. Biological processes that are overrepresented among the genes that are regulated in the *tpst-1* versus wild type. The identified genes were analyzed by the PANTHER16.0 programme (Mi *et al.*, 2019; Mi *et al.*, 2021). A total of 102 genes could be assigned to biological processes that are overrepresented.

Supplementary Figure S4

Gene / Comparison	*tpst-1* / wt	*tpst-1* + PSK / wt	*tpst-1* + PSK / *tpst-1*	Category
AT1G77640; member of the DREB subfamily A-5 of ERF/AP2 transcription factors	3.27	1.83	0.56	A
AT1G49960; Xanthine/uracil permease family protein	0.33	0.64	1.90	A
At3g22120; CWLP, Cell Wall-plasma Membrane Linker Protein	8.81	3.09	0.35	B
At5g35940; Mannose-binding lectin superfamily protein	0.06	0.29	5.24	B
At3g06019; unknown function	1.73	2.73	1.58	C
At3g21260; GLTP3, Glycolipid Transfer Protein 3	0.53	0.27	0.52	C

Supplementary Figure S4: Genes that could not be clearly categorized as regulated by PSK or other sulfated peptides and were sorted by different categories. Genes are derived from the microarray experiment. Microarray experiments were performed with three biological replicates.

Supplementary Figure S5

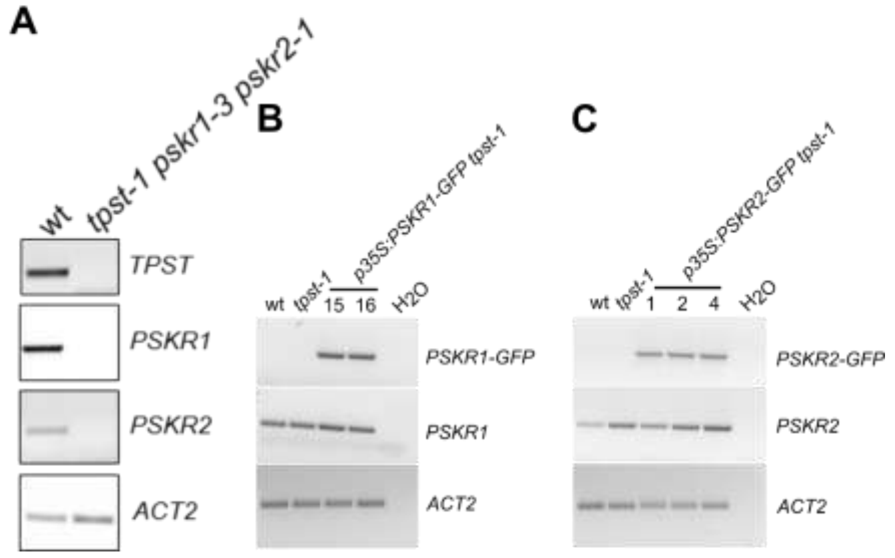

Supplementary Figure S5: Semi-quantitative RT-PCR analysis of **(A)** wt and *tpst-1 pskr1-3 pskr2-1*, **(B)** wt, *tpst-1* and two independent *p35S*:PSKR1-GFP *tpst-1* lines and (C) wt, *tpst-1* and three independent *p35S*:PSKR2-GFP *tpst-1* lines. Transcript levels of *TPST*, *PSKR1*, *PSKR1-GFP* and/ or *PSKR2* and *PSKR2-GFP* were analyzed in 5-day-old seedlings by RT- PCR. *Actin2* was amplified as a control for RNA input.

Supplementary Figure S6

Gene \ Comparison	*tpst-1* / wt	*tpst-1* + PSK / wt	*tpst-1* + PSK / *tpst-1*
At3g59900; ARGOS, Auxin-regulated gene involved in organ size	4.63	1.66	0.36
At5g06080; LBD33, Lob Domain-containing Protein 33	3.05	2.49	0.81
At1g76420; ANAC031, Arabidopsis Nac Domain Containing Protein 31	2.77	1.28	0.46
At5g12330; LRP1, Lateral Root Primordium 1	2.36	1.72	0.73
At3g27940; LBD26, Lob Domain-containing Protein 26	0.24	0.20	0.83

Supplementary Figure S6: Differentially regulated genes with functions in lateral root growth and development. Genes are derived from the microarray experiment. Microarray experiments were performed with three biological replicates.

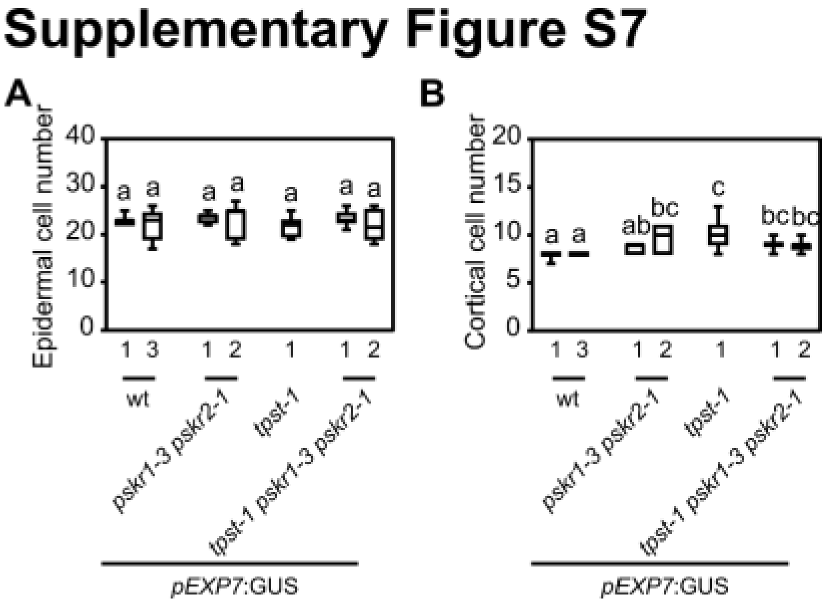

Supplementary Figure S7: Analysis of epidermal and cortical cell numbers (A, B) Quantification of (A) epidermal cell numbers and (B) cortical cell numbers in wild type, *pskr1-3 pskr2-1, tpst-1* and *tpst-1 pskr1-3 pskr2-1* that were determined from cross sections of *pEXP7:GUS*-expressing expressing lines. Numbers indicate independent transgenic lines. Experiments were performed at least three times with similar results. Data are shown for one representative experiment as the mean ± SE, (A) n ≥ 9, (B) n ≥ 9. Different letters indicate significant differences (Kruskal-Wallis, *P*<0.05).

Supplementary Table S1

Table S1: Primers used for analysis by PCR. For each gene, the upper sequence represents the forward primer, the bottom sequence the reverse primer.

locus	name	primer sequence (5' to 3')	analysis
At3g04120	*GAPC1*	GATTCTACAATGGCTGACAAGAAGA	qPCR
		ATGAAGGGGTCGTTGACAGC	qPCR
At3g18780	*ACT2*	ACATTCCAGCAGATGTGGATCTC	qPCR
		GATCCCATTCATAAAACCCCAGC	qPCR
At5g14750	*WER*	TCGTATTGCCAAAAAGACTGGTTTA	qPCR
		TGATAAGATCCTCTTCTTGCTCGG	qPCR
At5g40330	*MYB23*	CTCCTCGGCAACAGATGGTC	qPCR
		GGCTTTGACGGCAGTTGAAT	qPCR
At1g11130	*SCM*	AATCGGGGAAGGGTCGATTG	qPCR
		TTGAGGAATTCGCCGTCACT	qPCR
At1g66800		GCGATGAAGGCATGGTATGG	qPCR
		GCAAAACTGGTCCGATCACG	qPCR
At5g03150	*JKD*	ATGCGCAAGGTCTATCCGAG	qPCR
		AGGGTTTGTGGAAGTCATTGGA	qPCR
At1g12560	*EXP7*	TGCATACCGAAGAGTGCCAT	qPCR
		AACGGCCATGCTCTTGATGT	qPCR
At3g18780	*ACT2*	CAAAGACCAGCTCTTCCATCG	RT-PCR
		AGGTCCAGGAATCGTTCACAG	RT-PCR
At1g08030	*TPST*	GGCTCTTTTGCGGAACTTGA	RT-PCR
		CTTCAATTTTCGTGCATCTCG	RT-PCR
At2g02220	*PSKR1*	GTTTCGGAGTTGTGCTTCTCGAG	RT-PCR
		CCAAGAGACTAACTGTTGAGTCGTTG	RT-PCR
At5g53890	*PSKR2*	GAGGAGACTATCAGCGGGG	RT-PCR
		GGCCTAAGCAACCTCGCTAA	RT-PCR
GFP	*GFP*	forward primer from PSKR1 or PSKR2	RT-PCR
		CAGATGAACTTCAGGGTCAG	RT-PCR

At1g79840	*GL2*	CATGGACGTGGGACAATGGA	qPCR
		CATCAGCTGAATAGCCCCGT	qPCR
At1g12560	*EXP7*	TGCATACCGAAGAGTGCCAT	qPCR
		AACGGCCATGCTCTTGATGT	qPCR
At5g42600	*MRN1*	TGCATACACTTCCACCGCAT	qPCR
		GCTCCCAAACTGGCATACCT	qPCR
At5g42590	*MRO1*	CGGGCACACAGGTGATCATT	qPCR
		CGTCCAACAAAATCCCAAGTTGA	qPCR
At5g42580	*MRD*	TCGCTTTGTCCCAGTCAACA	qPCR
		GTGCATGTGGTCGGAGTAGT	qPCR
At4g15370	*BARS1*	CTCGACCATGGTGGTGCTAC	qPCR
		GAAGGAACCAGAACTCAGGGG	qPCR
At4g15360	*CYP705a3*	GGTGCAGTGCTTTGACTGGA	qPCR
		GGTTGGGTTCGAAGAACGGA	qPCR
At4g15350	*CYP705a2*	TGAATGAGGCTGCTGGAACA	qPCR
		AAGCAAGCTGACATCCCCAA	qPCR
At5g48010	*THAS*	GGTTAGAGTGGCTTAGTCCAGTG	qPCR
		CCCTGGAAACTGTTTGTTAAACTGA	qPCR
At5g48000	*THAH*	GTTACACAATTCCAGCGGGC	qPCR
		GCAACTCTTTCCCCTCCCAT	qPCR
At5g47990	*THAD1*	GAATGTCTCCTCTCGCCCTC	qPCR
		AGCAGCTTTTGGACCATGAAC	qPCR
